# Chicory (*Cichorium intybus* L.) and cereals differently affect gut development in broiler chickens and young pigs

**DOI:** 10.1186/2049-1891-4-50

**Published:** 2013-12-17

**Authors:** Haoyu Liu, Emma Ivarsson, Torbjörn Lundh, Jan Erik Lindberg

**Affiliations:** 1 Department of Animal Nutrition and Management, Swedish University of Agricultural Sciences, P.O. Box 7024, Uppsala SE-750 07, Sweden

**Keywords:** Broiler, Cereal arabinoxylans, Chicory uronic acids, Digestibility, Gut development, Microbiota, Pectin, Young pigs

## Abstract

Dietary fiber, resistant to host-mediated digestion in the small intestine due to lack of endogenous enzymes, impacts many facets of animal health and is associated with gut development especially in young monogastrics. Furthermore, it can be used as in-feed antibiotic alternative. Chicory (*Cichorium intybus* L.) forage with high content of pectin (uronic acids as building blocks) is a novel class of dietary fiber that is chemically different from cereal grains (with high content of arabinoxylans). In the present study, we investigated effects of dietary inclusion of chicory forage on digestibility, gut morphology and microbiota in broilers and young pigs. In the chicken experiment, 160 1-d old broiler chicks were fed 3 nutritionally balanced diets for 30 d including a cereal-based diet and 2 diets with part of the cereals substituted with 60 and 120 g/kg chicory forage (CF60 and CF120), whereas in the pig experiment, 18 seven-wk old Yorkshire pigs were fed 3 diets for 18 d including a cereal-based diet and 2 diets with 80 and 160 g/kg chicory forage inclusion (CF80 and CF160). Our results showed that young pigs were capable to utilize chicory forage well with higher total tract apparent digestibility (TTAD) of all fiber fractions, particularly uronic acid, compared with the control (*P* < 0.01). In contrast, a decreased TTAD of all fiber fractions was observed in chickens fed on diet CF120 (*P* < 0.05). Moreover, diet induced changes in gut morphology were observed in the large intestine of chickens. The alteration of cecal mucosal thickness was further positively correlated with TTAD of non-starch polysaccharides (NSP) and its constituent sugars (*P* < 0.05). In addition, in pigs, terminal restriction fragment length polymorphism (T-RFLP) analysis of intestinal microbiota revealed substantial dietary effects (cereal control diet vs. chicory forage inclusion) on the relative abundance of 2 dominant bacterial phylotypes (*Prevotella* sp. vs. *Roseburia* sp.) respectively (*P* < 0.05). In conclusion, our data showed that chicory forage (*Cichorium intybus* L.), a novel dietary fiber source in animal nutrition, have potential beneficial properties as fiber ingredient in diets for both pigs and chickens.

## Background

Dietary fiber is defined by CODEX Alimentarius as ‘carbohydrate polymers with 10 or more monomeric units, which are not hydrolyzed by the endogenous enzymes in the small intestine of humans’ [1]. This all-encompassing definition includes fiber naturally occurring in foods, as well as processed (physically, chemically or enzymatically) fiber from raw materials and synthetic fractions, in order to meet the needs of regulation and the associated labeling market. A substantial body of evidence demonstrates that fiber ingredients (mainly non-starch polysaccharides, NSP) constitutes an important component of a balanced diet and may affect many facets of animal nutrition and gut health, especially in young monogastrics [2-8]. The impact of dietary fiber on gut health opens a window in search for in-feed antibiotic alternatives. A prebiotic effect of dietary fiber will help to reduce the antibiotic usage in livestock and this will reduce the risk of transferring the antibiotic resistance gene to human pathogens [9]. Furthermore, dietary fiber has been associated with gut disorder management such as *Salmonella* infection in chickens and post-weaning diarrhea in pigs [10].

Arabinoxylan, composed of xylose as backbone and arabinose as side chains, is one major NSP fraction of the dietary fiber in cereal grains [2]. The arabinoxylans are present in both soluble and insoluble form, although a major part is insoluble. Cereals (*e.g.* wheat and barley) high in arabinoxylan content have been widely used in chicken and pig commercial feeds [6,11]. A study on humanized rats suggests that dietary arabinoxylan confers beneficial effects on gut health and may be a good candidate for prebiotics [2].

Chicory (*Cichorium intybus* L.) is a perennial herb that can produce nutritious and high quality forage [12]. The dietary fiber in chicory forage has high content of pectin (80–90 g/kg dry matter, DM), another type of NSP with uronic acid as building blocks, which is highly soluble in comparison with other pectin sources [8]. Chicory forage is a novel source of dietary fiber that has recently been shown to be well accepted and utilized by monogastric animals [8,13]. It is a potential feed resource that could partly replace cereal grain fiber in livestock feed and can be used as roughage source in organic pig farming [14]. Replacing cereal grain fiber may reduce feed cost and the conflict with human needs as there is limited supply of cereals and cereal by-products [15,16].

The present study investigated the impact of dietary inclusion of chicory forage on digestibility, gut morphology and gut microbiota in broiler chickens and young pigs. Our hypothesis was that chicory forage would affect the animals and their gut development differently from cereals.

## Methods

### Experimental design and animals

The animal experiment was performed at the Swedish University of Agricultural Sciences (SLU) in Uppsala and was approved by the ethical committee for the Uppsala region. The chicken trial was structured as a randomized block design with 3 treatments, 4 blocks for control group and 2 × 8 blocks for experimental groups, respectively. Each block contained 8 birds. In the experiment using young pigs, a split-litter design was used with 18 pigs from 6 different litters (3 pigs per litter). Animals were randomly distributed to one of 3 treatments (6 replicates for each).

In the chicken trial, 160 female and male 1-d-old broiler chicks (Ross 308) with an initial body weight (BW) of 44.0 ± 15.1 g were studied over a 30-d period. The birds were kept on wood shaving floors from d 1 to d 27. Thereafter, a net floor was used for 3 d for excreta sampling. Chickens had free access to feed and water throughout the experiment. The environment temperature and light were strictly controlled. Body weight and feed intake were recorded for each pen on the first d of the experiment and every wk thereafter.

In the pig trial, 18 seven-wk old Yorkshire pigs (castrated male and female) with an initial BW of 11.7 ± 0.16 kg were studied over an 18-d period. The pigs were housed in individual pens without straw bedding and supplied with feed and water *ad libitum*. Feed intake was recorded daily. The BW was registered weekly and on the last d of the experiment.

### Diets

Chicory forage (*Cichorium intybus* L.) was used to compose the experimental diets and replaced the cereal fraction (wheat and barley) in the cereal control diet. All diets were supplemented with protein, amino acids, minerals, and vitamins to meet the nutritional requirements of the broilers and the growing pigs, respectively [17,18]. However, in order to keep diet composition constant throughout the experimental period, the crude protein (CP) content for chickens was lower than recommended during the first wk. Prior to mixing with other feed ingredients, chicory forage was dried at low temperature (30°C) with forced air for a wk. All ingredients were milled through a 3-mm screen for chicken feed and a 3.5-mm screen for pig feed and were fed as pellets to the animals in all cases except for the first wk for chicks, when pellets were ground. Titanium oxide (TiO_2_) was included in the diets as an internal digesta marker. No antibiotics were administered.

The detailed diet ingredient composition is shown in Table 1. In brief, for the chicken the experimental diets were comprised of the cereal-based basal diet (C^I^) and diets with inclusion of 60 and 120 g/kg chicory forage (CF60 and CF120). For the young pigs, the experimental diets were comprised of the cereal-based basal diet (C^II^) and diets with inclusion of 80 and 160 g/kg chicory forage (CF80 and CF160).

**Table 1:** Diet ingredients (g/kg), analyzed chemical composition (g/kg DM) and gross energy content (MJ/kg DM) of control and experimental diets

	**Chicken diets**			**Young pig diets**		
**Items**	**C**^**I**^	**CF60**	**CF120**	**C**^**II**^	**CF80**	**CF160**
Wheat	550	507.5	460	410	370	320
Barley	187.5	170	160	400	360	330
Protein sources	160	160	160	160	160	160
Vegetable fat	30	30	30	10	10	10
Chicory	0	60	120	0	80	160
Premix	2	2	2	4	4	4
Titanium oxide	5	5	5	2.5	2.5	2.5
Others	65.5	65.5	63	13.5	13.5	13.5
Dietary fiber	171	183	196	137	175	196
Klason lignin	19	23	21	27	38	43
NSP	152	160	175	110	137	153
Arabinose	24	26	22	18	19	19
Xylose	40	38	34	37	36	36
Uronic acid	18	27	37	4	17	24
Fructan	20	21	18	8	10	6
Gross energy	18.0	18.0	18.0	18.7	18.4	18.2

C^I^: control diet in chicken experiment; CF60: 60 g/kg chicory forage; CF120: 120 g/kg chicory forage; C^II^: control diet in pig experiment; CF80: 80 g/kg chicory forage; CF160: 160 g/kg chicory forage.

For broiler diets, soybean meal was used as protein sources, whereas for young pig diets, a mix of fish meal and potato protein was used.

Premix in chicken experiment: vitamin A, (all-trans retinol) 12000 IU; vitamin D_3_, 5000 IU; vitamin E, (DL-α-tocopheryl acetate 70 IU); vitamin K_3_, 4 mg; vitamin B_1_, 3 mg; vitamin B_2_,. 8 mg; vitamin B_6_, 5 mg; vitamin B_12_, 0.02 mg; Pantothenic acid, 20 mg; Folic acid, 2 mg; Niacin, 60 mg; Biotin, 0.175 mg; Iron, 20 mg; Copper, 15 mg; Cobalt, 0.25 mg; Manganese, 70 mg; Zink, 70 mg; I, 1 mg; Selenium, 0.035 mg; Molybdenum, 0.50 mg. Premix in pig experiment: Ca 3.77 g, P 1.02 g, Mg 3.10 g, K 1.50 g, Na 0.37 g, Cl 0.11 g, S 84.00 g, Fe 120493 mg, Cu 29791 mg, Co 52.62 mg, Mn 24076 mg, Zn (as ZnO4) 134 mg, I 238 mg, Se 476 mg. Vitamins: retinol 5952000 IE, cholecalciferol 595200 IE, α-tocopherol 101190 mg, thiamin 2381 mg, riboflavin 5952 mg, pyroxidine 3750 mg, B_12_ 24 mg, pantothenic acid 11914 mg, nicotinic acid 23810 mg, biotin 2238 mg.

Others in chicken experiment(g): NaCl_3_, L-lysine 3.5-4, DL-methionine, 4–4.5, monocalciumphosphate, 17.5-18, calcium carbonate, 19.5-20; Others in pig experiment (g): NaCl 2.5, _L_-lysine-HCL 0.5, _DL_-methionine 0.5, monocalciumphosphate 5, limestone 5.

### Sampling and analysis

Feed and feces were collected for chemical analysis. At the end of the animal experiment, chickens were killed by an intravenous injection of sodium pentobarbital through the wing vein, whereas pigs were sedated first and killed by a lethal dose of pentobarbital sodium (60 mg/mL; Apoteket, Umeå, Sweden) at 100 mg/kg BW. For histological analysis, intestinal tissues were sampled as previously [13]. In chickens, jejunum and cecum were sampled, whereas in pigs were distal ileum and proximal colon taken, representing the small and large intestine respectively. In addition, in the pig experiment, intestinal digesta were collected correspondingly.

The chemical composition and gross energy of diets were analyzed as previously described [19] and are shown in Table 1. Gross energy was measured with a bomb calorimeter (Parr 6300 Oxygen Bomb Calorimeter, Illinois, USA). TiO_2_ was used for calculation of total tract apparent digestibility (TTAD) of dietary components [20].

Intestinal histological parameters, including villus height and crypt depth in the small intestine and mucosal thickness in the large intestine were determined according to standard procedures: villus height was depicted from its apex to the transition into the crypt zone, whereas crypt depth was measured as the difference between mucosal thickness (the distance from villus top to crypt end) and villus height.

In order to explore dietary fiber impact on the animal gut and associated changes of microbiota composition, terminal restriction fragment length polymorphism (T-RFLP) analysis was carried out on intestinal digesta samples from pigs as previously described [21]. Putative identifications of TRFs were obtained by ribosomal database mining (http://mica.ibest.uidaho.edu/) and by comparisons with our internal database (based on T-RFLP analysis following 10 clone libraries constructed from our previous pig studies). Moreover, the relative abundance of TRFs within a microbial community profile was used to determine the bacterial diversity [21].

### Statistical analysis

Statistical analyses were performed with various procedures in SAS (SAS Institute, Cary, NC, USA, version 9.2). Dietary effects were analyzed using PROC GLM in chicken experiment and PROC MIXED in pig experiment. Furthermore, PROC CORR was carried out to identify relationships between variables. Data were presented as least square means ± SEM. Significance was set at *P* < 0.05.

## Results and discussion

Cereal arabinoxylan and plant-origin pectin are NSP abundant in animal diets. Although resistant to host-mediated digestion in the small intestine, these substrates can serve as energy source and physiological stimuli for gut development and microbiota modulation in the large intestine [6,11,22]. In the present study, animals maintained feed intake and growth rate irrespective of dietary treatments [6,19], except that during the first 2 wk of rearing period for chickens, the growth rate was decreased on diet CF120 as compared with the breed standard [23]. This may be attributed to the single feed usage throughout the experiment (lower CP content for younger birds). Overall, the present data on chickens and pigs performance are in the same range as in previous studies using various dietary fiber sources [5,7,11,24].

### Dietary fiber utilization in chickens and young pigs

The digestibility of dietary fiber is highly variable and is related to its origin [15]. The present study focused on different chemical composition of fiber fractions in the diets, in which pectin (uronic acid as building blocks) was a large NSP component in the chicory diets differently from the control diet (Table 1). In chickens, the average TTAD of total NSP was 28.4%, which decreased with increasing fiber inclusion level, ranging from 23.8% on diet CF120 to 31.2% on diet C^I^ (Table 2; *P* = 0.008). A similar response was also detected for the TTAD of uronic acid (*P* = 0.02), arabinose (*P* = 0.0002) and xylose (*P* = 0.0007), which agreed with results from studies on chickens fed with pea fiber [25]. In contrast, the TTAD of all fiber components increased in pigs with dietary chicory inclusion (*P* < 0.05) (Table 2). Apparently, growing pigs were able to digest chicory fiber to a greater extent than chickens. Ivarsson and co-workers (2011) have reported a TTAD of total NSP of 67% in pigs fed diets with chicory forage inclusion [8]. Intriguingly, the most completely digested NSP fraction in the present study was uronic acid (building blocks of pectin). The TTAD of uronic acid was on average 41.5% in chickens and 70.0% in pigs. Thus, this suggests that chicory forage should be classified as a highly digestible and palatable fibrous feed ingredient for chicken and pigs. However, high inclusion levels of chicory forage (120 g/kg) for broilers may be a challenge [6].

**Table 2:** Effects of diets on total tract apparent digestibility (TTAD) of fiber in chickens and pigs

	**In chickens**					**In pigs**				
		**Diet**					**Diet**			
**Items**	**C**^**I**^	**CF60**	**CF120**	**SEM**	***P-*** **value**	**C**^**II**^	**CF80**	**CF160**	**SEM**	***P-*** **value**
Total NSP^*^	31.2^a^	30.1^a^	23.8^b^	1.65	0.008	42.0^a^	56.1^b^	62.4^c^	1.42	<0.0001
Uronic acid	43.3^a^	48.0^a^	33.1^b^	3.34	0.02	36.2^a^	85.1^b^	88.8^b^	1.47	<0.0001
Arabinose	31.0^a^	34.3^a^	18.4^b^	2.40	0.0002	46.8^a^	58.0^b^	62.8^b^	1.71	<0.0001
Xylose	28.5^a^	23.8^a^	16.5^b^	1.77	0.0007	39.8^a^	45.7^b^	49.4^b^	1.70	0.004

C^I^: control diet in chicken experiment; CF60: 60 g/kg chicory forage; CF120: 120 g/kg chicory forage; C^II^: control diet in pig experiment; CF80: 80 g/kg chicory forage; CF160: 160 g/kg chicory forage. *NSP, non-starch polysaccharides.

Data are presented as least square means (n = 4 for diet C^**I**^and 8 for diet CF60 and CF120 in chicken experiment; n = 6 in pig experiment) and standard error of the mean (SEM).

^abc^Different letters within rows, indicate significant difference (*P* < 0.05).

### Dietary fiber–induced changes in gut morphology

Animal performance and digestive capacity are suggested to be interrelated with gut development [22,26]. Indeed, fiber utilization is more limited in chickens than in pigs, largely due to its shorter foregut and a higher digesta passage rate than in pigs [10]. We therefore speculated that utilization of the fiber fraction in response to dietary chicory inclusion would also be manifested at gut level in chickens and pigs. We assessed gut morphometry in the small and large intestine and its relationship with TTAD of dietary fiber components. The small intestinal morphology was not altered by diets (Table 3; *P* > 0.05), whereas the large intestinal morphology in chicken’s was (*P* = 0.001). This may be attributed to the fact that fiber degradation mainly took place in the large intestine [10]. Furthermore, positive correlations were identified between mucosal thickness in the large intestine and TTAD of NSP (Table 4; r = 0.44; *P* = 0.05) and its constituent sugars in chickens (*P* < 0.05). The present results in chickens suggest that it is the extent to which the dietary fiber is digested in the gut that will affect the intestinal morphology rather than the fiber inclusion level in diet *per se*. In contrast, no diet responses or association with diet digestibility was identified in young pigs. Possibly, the porcine gut is less sensitive to dietary fiber ingestion than the gut of chickens [10]. Moreover, the experimental period was only 18 d in pigs compared with 30 d in broiler chickens. Thus, the time of gut exposure to the fiber sources could be the major reason for the species differences in intestinal morphology changes.

**Table 3:** Dietary induced changes in gut morphology of broilers and pigs

	**In chickens**					**In pigs**				
**Item**		**Diet**					**Diet**			
	**C**^**I**^	**CF60**	**CF120**	**SEM**	***P-*** **value**	**C**^**II**^	**CF80**	**CF160**	**SEM**	***P-*** **value**
Small intestine VH	1380	1408	1379	55.8	NS	814	833	806	43.4	NS
Small intestine CD	171	170	161	16.4	NS	265	265	257	18.5	NS
Large intestine MT	376^a^	310^b^	285^b^	13.0	0.001	538	551	524	20.2	NS

C^I^: control diet in chicken experiment; CF60: 60 g/kg chicory forage; CF120: 120 g/kg chicory forage; C^II^: control diet in pig experiment; CF80: 80 g/kg chicory forage; CF160: 160 g/kg chicory forage. VH, villus height; CD, crypt depth; MT, mucosal thickness; NS, non-significant.

Data are presented as least square means (n = 4 for diet C^**I**^and 8 for diet CF60 and CF120 in chicken experiment; n = 6 in pig experiment) and standard error of the mean (SEM).

^ab^Different letters within rows, indicate significant difference (*P* < 0.05).

**Table 4:** Correlations between mucosal thickness and total tract apparent digestibility (TTAD) of fiber components in chickens

**Variable**	**With variable**	**r**	***P*-value**
Mucosal thickness	NSP TTAD	0.440	0.05
Mucosal thickness	Xylose TTAD	0.538	0.01
Mucosal thickness	Uronic acid TTAD	0.519	0.02

Data are presented as least square means and standard error of the mean (SEM). Significance was set at *P* < 0.05.

### Dietary fiber-induced changes in gut microbiota in young pigs

We assumed that the microbiota in the large intestine of our pigs would be shifted by diet intervention, especially by fiber content variation, in a short period of time. This was based on observations in humans, showing that changing the diet from high-fat/low-fiber to low-fat/high-fiber alters the gut microbiota composition within 24 h [27]. Thus, we hypothesized that diets rich in chicory or cereal fiber would result in microbiota of very different species composition.

**Figure 1:**
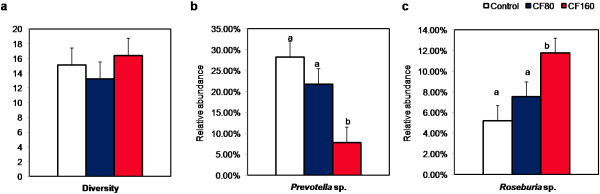
**Effects of diet on bacterial diversity (a) and relative abundances of phylotypes corresponding to *Prevotella *sp. (b) and *Roseburia *sp. (c) in the large intestine of pigs. CF80: 80 g/kg chicory forage; CF160: 160 g/kg chicory forage.** Data are presented as least square means (n = 6) and standard error of the mean (SEM). ^ab^ Different letters within rows, indicate significant difference (*P* < 0.05).

T-RFLP analysis was done to compare bacterial profile in pigs fed on different diets. In addition, Simpson’s index of diversity was calculated based on T-RFLP data to address the bacterial species richness and evenness in the gut ecosystem [21]. However, no significant difference between diets was found on bacterial diversity in the present study (Figure 1a, *P* > 0.05). This was at least partly due to the large individual variation of microbiota composition in our pigs (data not shown), which is in agreement with previous mapping of the porcine gut microbial ecology [28]. In this study, Leser and co-workers (2002) demonstrate that with 400 intestinal bacterial phylotypes identified in pigs, more than 90% have fallen into two phyla, i.e., *Firmicutes* and *Bacteroidetes*. Moreover, members of the *Firmicutes* are most represented by clostridia species, whereas *Prevotella* spp. is the most abundant *Bacteroidetes* bacteria group in the porcine large intestine.

Interestingly, we found that the most dominant TRFs in our pig gut was TRF264, followed by TRF274, which corresponded to one species of *Prevotella* sp. (Figure 1b) and *Roseburia* sp. (bacteria belong to clostridial cluster XIVa; Figure 1c), respectively. Substantial dietary effects on these 2 dominant bacterial phylotypes were revealed. The relative abundance of *Prevotella* sp. was highest on the cereal-based control diet, and decreased with more cereals substituted by chicory forage (*P* = 0.004). This may be because *Prevotella* species are capable of producing enzymes such as xylanases, mannanases, β-glucanase, *etc.* that can degrade dietary xylans in the large intestine [29]. In contrast, changes of the second dominant bacterial phylotype *Roseburia* sp. in our pigs was driven by chicory fiber (with high content of uronic acids), as a marked increase in the relative abundance was observed with increasing inclusion of chicory forage in the diet (*P* = 0.004). Clostridial species have been suggested to play a central role in bacterial cross feeding in the gut microenvironment, converting acetate and lactate to butyrate [30]. Identification of interactions between certain NSP fractions and such bacteria in the gut is of great importance, yet very limited information is available [31]. For pectin in particular, there is a lack of data on its utilization by different microbes. One recent study showed that a member of clostridial bacteria is able to outgrow other microbes on apple pectin *in vitro,* indicating the essential role pectin may play as a substrate for this bacterial group [32]. However, questions remain whether all intestinal *clostridium* species can utilize dietary pectin and whether a common metabolic pathway is shared.

## Conclusions

In conclusion, we have demonstrated that chicory (*Cichorium intybus* L.) forage can be used as a highly digestible and palatable fibrous feed ingredient in chicken and pig nutrition. Inclusion of chicory, high in pectin, affects gut morphology and gut microbiota community composition differently from cereal fiber. Furthermore, we found that the extent to which the NSP fractions were digested played a major role for the gut morphology in chickens and not the fiber inclusion level *per se*.

## Abbreviations

NSP: Non-starch polysaccharides; DM: Dry matter; BW: Body weight; CP: Crude protein; TiO2: Titanium oxide; TTAD: Total tract apparent digestibility; T-RFLP: Terminal restriction fragment length polymorphism.

## Competing interests

The authors declare that they have no competing interests.

## Authors’ contributions

JEL and TL conceived the study and experimental design. HYL and EI carried out the experiment trials. HYL and EI performed lab analysis. HYL performed the statistics and drafted the manuscript. JEL revised initial draft manuscript. All authors read and approved the final manuscript.
